# Viburnum Prunifolium as a Uterine Sedative

**Published:** 1878-02

**Authors:** 


### Selections.

---

VIBURNUM PRUNIFOLIUM AS A UTERINE SEDATIVE.—It was not my intention to bring this remedy before the notice of the society until my own experience in the use of it was much larger than at present, but as it has proved so very satisfactory and efficacious in the cases in which I have used it during the past two months, probably others who have more frequent opportunities than myself may be induced to give it a trial, and report to the society, at some future meeting, the result of their experience with it.

In the following cases, the viburnum was used with benefit:

October 28, 1877, 9.30 P.M.—Mrs. D., aged 28, last child 18 months old, which she continues to nurse, menstruated last from 24 to 28 august, was taken with pains and uterine hemorrhage about 6 o'clock in the evening. Os dilated sufficiently to allow one finger to pass; blood came freely in gushes at intervals of about three minutes. Ordered half a teaspoonful of the fl. ex. viburnum every half hour until hemorrhage was checked. After the third dose there was no more hemorrhage, it having gradually diminished after the first dose. She continued one-half teaspoonful three times daily for three or four days.

October 17, 1877.—Mrs. R. C., in the seventh month of pregnancy, had been flooding profusely for four or five days. Ordered fluid extract viburnum ʒi to be taken at once, ʒss every half hour until checked. The hemorrhage was entirely checked after taking a few doses.

In two cases of menorrhagia, in which the menses had always lasted seven or eight days, they were decreased to five days, and were much diminished in quantity. In these cases the viburnum was taken in ʒss doses three times daily.

September 14, 1877.—Mrs. H. E. B., aged 30, in the seventh month of her second pregnancy, for the past two months has had almost constant headache, associated with insomnia, impaired vision and vertigo. Œdema of the face and of the upper and lower extremities, frequent nausea and vomiting, mouth and tongue very sore and excessive ptyalism—urine highly albuminous. Was treated for the albuminuria, which gradually diminished, and in about a month was altogether absent. She was now entirely relieved from the headache, vertigo and œdema, but the ptyalism, sore mouth and tongue were, if anything, even worse than when I first saw her. None of the usual mouth washes for this complaint seemed to give her any relief. She used chlorate of potash, borax, bismuth, slippery elm infusion, emulsion of bitter almonds with hydrocyanic acid, belladonna, etc.

About the first of November she commenced taking ℨss of the fluid extract viburnum every three hours. Her mouth soon commenced to get better, and in less than a week gave her no more trouble.

My attention was first called to this remedy by a paper which I had the pleasure of hearing read by Dr. Edward W. Jenks, of Detroit, Michigan, before the American Gynæcological Society, in New York, in September, 1876.

Dr. Jenks said he had used the viburnum during the past ten years in a great number of cases of threatened abortion, and that he now relies upon it as the most efficacious remedy we have for this trouble. He recommends it especially in those cases where abortion has become habitual with a woman. He also recommends it as a valuable therapeutic agent in the treatment of the sympathetic disorders incident to pregnancy, where a nervine or sedative is demanded. He also says that he has found it extremely useful in a large class of non-puerperal diseases of women, especially in all uterine disorders characterized by loss of blood, such as menorrhagia, metorrhagia incident to the menopause, and in all forms of dysmenorrhœa attended with profuse menstruation.

The attention of the profession was first called to the uses of the viburnum by an article written by Dr. Phares, of Newtonia, Mississippi, which was first published in the Atlanta Medical and Surgical Journal, in 1866, and subsequently republished in the Detroit *Review*, December, 1866, and in the Boston *Medical and Surgical Journal*, October 10, 1867.

Dr. Phares attached particular value to this remedy for the prevention of abortion. He designated it as a "nervine, antispasmodic, tonic, astringent and diuretic," and adds: "But it is particularly valuable in avoiding abortion and miscarriage, whether habitual or otherwise, whether threatened from accidental cause or criminal drugging. It tones up the system, preventing or remov ing those harassing nervous symptoms that so often torment and wear down the pregnant woman, and disqualify her for the parturient effort. It enables the system to resist the deleterious influence of drugs so often used for the purpose of procuring abortion."

He also alludes to the habit, common among negro women on many of the Southern plantations, of taking a decoction of gossypium, or cotton root, for the purpose of procuring abortion, and says: "Some farmers on whose plantations I have used the medicine, and who have seen much of its effect on negro women who had always managed to miscarry, declare their belief that no woman can possibly abort if compelled to use the viburnum."

Of course, no intelligent physician expects that when an abortion is fairly begun by detachment of the ovum, or when a portion of it is extruded from the uterus, any remedy or treatment will prevent its ultimate expulsion; the mischief is already done; the vital connection of the fœtus with the mother is destroyed, and no measure can preserve its life.

In order to determine in what cases this drug acts as a means for preventing abortion, we may very properly consider the causes of abortion under the four following heads:

1. *Those that are accidental.*—Violent mental emotion; sudden agitation from fright; great bodily fatigue, with mental anxiety and severe pain; hysterical convulsions, blows, falls, irritation of mammary nerves, lactation and constant suckling, railway traveling, drastic purgatives, placenta prævia, etc.

2. *Those that are due to some deranged state of the mother's health.*—Acute thoracic and abdominal diseases, albuminurea, uræmic convulsions, measles, scarlatina, small-pox, obstinate constipation, syphilis, intermittent fever, etc.

3. *Those that can be traced to some morbid condition of the uterus or its appendages.*—Adhesions of the uterus to other pelvic organs, retroversion of gravid uterus, etc.

4. *Those that arise from disease of the embryo or its membranes.*—Small-pox, cholera, scarlet fever, hydrocephalus, knotting or compression of the funis, placentitis, fatty degeneration, hypertrophy, induration, calcification or ossification of the placenta, and syphilis.

It is in the first class, those that are accidental, that viburnum, by arresting the contractions of the uterus, which serve to separate the utero-placental attachments, and by modifying the placental and utero-placental circulation, acts so favorably as a uterine sedative in preventing threatened abortions. In the second and third classes, it might be a valuable adjuvant to other appropriate treatment, especially in cases of retroversion of the gravid uterus. But in the fourth class, where the abortion is caused by disease of the embryo or its membranes, such as small-pox, scarlet fever, hydrocephalus, knotting or compression of the funis, fatty degeneration of the placenta or syphilis, the fœtus is generally dead some time previous to the threatened abortion, and of course no treatment at this stage can be expected to arrest it.—*Maryland Medical Journal.*

